# Pathological Glucose Levels Enhance Entry Factor Expression and Hepatic SARS‐CoV‐2 Infection

**DOI:** 10.1111/jcmm.70581

**Published:** 2025-05-29

**Authors:** Guocheng Rao, Xiongbo Sang, Xinyue Zhu, Sailan Zou, Yanyan Zhang, Wei Cheng, Yan Tian, Xianghui Fu

**Affiliations:** ^1^ Department of Endocrinology and Metabolism, Department of Biotherapy, Center for Diabetes and Metabolism Research, State Key Laboratory of Biotherapy and Cancer Center, West China Hospital Sichuan University Chengdu Sichuan China; ^2^ Department of Endocrinology and Metabolism Gansu Provincial Hospital Lanzhou China; ^3^ Division of Pulmonary and Critical Care Medicine, State Key Laboratory of Biotherapy and Cancer Center, West China Hospital Sichuan University Chengdu Sichuan China

**Keywords:** COVID‐19, diabetes, glucose‐lowering drugs, metabolic dysregulation, NAFLD

## Abstract

Accumulating clinical evidence suggests an intricate relationship between severe COVID‐19 and preexisting metabolic complications, which share some metabolic dysregulations, including hyperglycaemia, hyperinsulinaemia and hyperlipidaemia. However, the potential role of these metabolic risk factors in SARS‐CoV‐2 infection and entry factor expression remains unknown. Here we report the implication of hyperglycaemia in SARS‐CoV‐2 infection and therapy. Hyperglycaemia, instead of hyperinsulinaemia and hyperlipidaemia, can significantly induce the expression of SARS‐CoV‐2 entry factors (*Ace2*, *Tmprss2*, *Tmprss4*, *Furin* and *Nrp1*) in liver cells, but not in lung and pancreatic cells, which is attenuated by mTOR inhibition. Correspondingly, pathological glucose levels promote SARS‐CoV‐2 entry into cultured hepatocytes in pseudovirus cell systems. Conversely, representative glucose‐lowering drugs (metformin, dapagliflozin, sitagliptin and exenatide) are able to diminish the enhancement of entry factor expression and SARS‐CoV‐2 infection in cultured hepatocytes under pathological glucose conditions. Intriguingly, SARS‐CoV‐2 entry factors are increased in the livers of nonalcoholic fatty liver disease and diabetes patients. These results define hyperglycaemia as a key susceptibility factor for hepatic SARS‐CoV‐2 infection, and provide insights into the clinical application of glucose‐lowering therapies in COVID‐19 patients under comorbid hyperglycaemia conditions.

**Abbreviations**
ACE2: angiotensin‐converting enzyme 2
COVID‐19: coronavirus disease 2019
DPP4: dipeptidyl peptidase‐4
FURIN: furin, paired basic amino acid cleaving enzyme
GLP‐1: glucagon‐like peptide‐1
mTOR: mammalian target of rapamycin
NAFLD: nonalcoholic fatty liver disease
NRP1: neuropilin‐1
PA: palmitic acid
RG4: RNA G‐quadruplex
SARS‐CoV‐2: severe acute respiratory syndrome coronavirus 2
SGLT2: sodium‐glucose cotransporter‐2
TMPRSS2: transmembrane serine protease 2
TMPRSS4: transmembrane serine protease 4
VSV: vesicular stomatitis

## 1 Introduction

Coronavirus disease 2019 (COVID‐19), caused by the SARS‐CoV‐2 virus, first emerged in December 2019. Despite the development of vaccines and improved therapies, global infections still spread at an alarming rate. Owing to its high contagiousness, the occurrence of asymptomatic carriers, and the rise of viral variants, SARS‐CoV‐2 remains a devastating threat to human lives, the global economy and society [1]. In this regard, prevention before disease onset, as well as the combination of prevention and cure, is vitally important to beat the COVID‐19 pandemic. Therefore, it is of significance and urgency to identify and understand the pathogenesis of SARS‐CoV‐2 infection, especially the viral entry step, which enables the development of viable interventions to prevent COVID‐19, complementing available vaccination efforts.

SARS‐CoV‐2 relies on cellular host factors to fulfil its life cycle, such as entry, replication and dissemination [2, 3]. Specifically, SARS‐CoV‐2 entry into human cells is initiated by the binding of the viral Spike protein with the angiotensin‐converting enzyme 2 (ACE2) receptor on the surface. Then, certain host proteases, particularly TMPRSS2 (transmembrane serine protease 2) and FURIN (furin, paired basic amino acid cleaving enzyme), are required for cleavage activation of the Spike protein, facilitating membrane fusion and viral entry into the host cytosol. Besides TMPRSS2, TMPRSS4 may also serve as a serine protease to promote Spike protein cleavage and SARS‐CoV‐2 entry [4]. In addition, neuropilin‐1 (NRP1), known to bind furin‐cleaved substrates, can enhance the interaction of SARS‐CoV‐2 with ACE2, and thus potentiates virus entry and infectivity [5, 6]. Consistent with the function of entry factors, their dysregulation has been implicated in SARS‐CoV‐2 infection and pathogenesis. For example, we have recently shown that *Tmprss2* is suppressed by RNA G‐quadruplex (RG4), a non‐canonical RNA secondary structure, and RG4 stabilisers can attenuate SARS‐CoV‐2 infection [4, 7]. Currently, cellular host factors are considered attractive targets for prevention and treatment of COVID‐19 [2]. To achieve this goal, it is fundamental to understand the mechanisms underlying the dynamic regulation of host factors in COVID‐19 pathophysiology.

Accumulating evidence indicates that a number of comorbidities, particularly obesity, type 2 diabetes and cardiovascular disease, are associated with worse COVID‐19 outcomes [8, 9, 10, 11]. Notably, nearly 50% of COVID‐19‐related deaths are attributed to patients with preexisting vascular and metabolic diseases. Interestingly, many COVID‐19 comorbidities share certain metabolic risk factors, such as insulin resistance and dysregulation of glucose and lipids [12, 13, 14, 15]. Therefore, unprecedented research efforts have recently been spurred to explore the relationships and interactions between dysmetabolism and poor COVID‐19 outcomes. Of note, there is a little data that hint that aberrant metabolism might affect the SARS‐CoV‐2 life cycle and infection. For instance, elevated circulating glucose levels can promote SARS‐CoV‐2 replication and proinflammatory cytokine production in monocytes, thereby favouring virus infection and severe COVID‐19 symptoms [16]. However, the potential role of metabolic risk factors in SARS‐CoV‐2 entry factor expression and SARS‐CoV‐2 infection has not been investigated.

Herein, we report a role of hyperglycaemia in SARS‐CoV‐2 infection and pathogenesis. Hyperglycaemia, instead of hyperinsulinaemia and hyperlipidaemia, can significantly induce the expression of SARS‐CoV‐2 entry factors in the liver, but not in the lung and pancreas, which is attenuated by the inhibition of mechanistic target of rapamycin kinase (mTOR). Correspondingly, pathological glucose levels enhance SARS‐CoV‐2 entry into cultured hepatocytes in pseudovirus cell systems. Conversely, glucose‐lowering drugs can attenuate the enhancement of entry factor expression and SARS‐CoV‐2 infection induced by elevated glucose levels. In addition, SARS‐CoV‐2 entry factors are generally increased in the livers of hyperglycaemia‐related diseases. These findings define a metabolic mechanism for hepatic SARS‐CoV‐2 susceptibility and suggest potential strategies for COVID‐19 prevention and treatment.

## 2 Materials and Methods

### 2.1 Human Studies

All human studies were conducted according to the principles of the Declaration of Helsinki, and approved by the Institutional Review Board and Biomedical Ethics Committee of West China Hospital of Sichuan University (WCH/SCU). The biobanking procedures were certified by the China Human Genetic Resources Management Office as a part of West China Biobanks (2016, no. 406). Fully informed consent was obtained from all patients prior to the sampling of liver tissue. The procedures for clinical data collection were described in our previous studies [17, 18] and data were stored in an electronic database (Microsoft Access).

### 2.2 Cell Culture and Treatment

Human lung (A549 and H1299), hepatic (Huh7 and HepG2) and pancreatic (MIA PaCa‐2 and Panc‐1) cell lines were chosen for this study, based on their extensive use and their relevance to COVID‐19‐associated biological processes [19, 20]. A549, HepG2, MIA PaCa‐2 and PANC‐1 cells were purchased from the American Type Culture Collection. Huh7 and H1299 cells were obtained from the Cell Bank of Type Culture Collection of Chinese Academy of Sciences (Shanghai, China). All cells were cultured in Dulbecco's modified Eagle's medium (DMEM; Gibco, USA) supplemented with 10% fetal bovine serum (FBS; Gibco, USA) and 1% penicillin–streptomycin (Gibco, USA). For glucose treatment, cells were cultured with 5.5, 25 and 100 mM glucose, respectively, for 48 h [21]. For insulin stimulation, cells were starved for 6 h and then cultured in DMEM medium containing 10 nM insulin for 12 h. For mimicking hyperlipidaemia, cells were cultured in DMEM medium containing 250 μM palmitate for 24 h.

For rapamycin treatment, cells were cultured with 5.5, 25 and 100 mM glucose, respectively, for 24 h, and then incubated with DMEM medium containing 2 μM rapamycin under different glucose concentrations for 48 h. For gene knockdown, cells incubated in different glucose concentrations (5.5, 25 and 100 mM) were transfected with indicated siRNAs using HiPerFect transfection reagent (Qiagen, no. 301705) for 48 h as described previously [22]. The sequences of siRNAs are shown in Table S1.

For glucose‐lowering drugs treatment, cells were cultured with different glucose concentrations (5.5, 25 and 100 mM) for 24 h, and then incubated in medium containing dapagliflozin (0.5 mM; Selleck, no. S5566), exenatide (100 nM; MCE, no. HY‐13443), metformin (50 mM; MCE, no. HY‐17471A) or sitagliptin (600 nM; Selleck, no. S4002) for 24 h.

### 2.3 RNA Extraction and Quantitative Real‐Time PCR

Total RNA isolation and quantitative real‐time PCR (qPCR) were performed as described previously [23]. Briefly, total RNA was extracted using TRIzol reagent (MRC, no. TR118), and then 1–2 μg RNA was used for reverse transcription into cDNA by using M‐MLV Reverse Transcriptase (Thermo Fisher Scientific, no. 28025021). qPCR was conducted using SYBR Green PCR Master Mix (Qiagen, no. 208052) with specific primers. Primer sequences for qPCR are described in Table S2.

### 2.4 Western Blotting

Total protein extraction and western blotting were performed as described previously [24]. Briefly, cells and tissues were homogenised on ice in RIPA lysis buffer (Thermo Fisher Scientific, no. 89901) with protease inhibitors (Roche, no. 11873580001) and phosphatase inhibitors (Bimake, no. B15001). Protein concentration was quantified by the Bradford assay (Bio‐Rad, no. 500‐0205) and an equal amount of total protein was used for denaturalization. The denatured samples were resolved by SDS‐PAGE and then transferred to a PVDF membrane (GE, no. A10122278). Subsequently, the membrane was blocked with non‐fat milk, incubated with primary antibodies followed by horseradish peroxidase (HRP)‐conjugated secondary antibodies, and then developed using the Pierce SuperSignal system (Thermo Scientific, no. 34096). Antibodies used in western blotting were listed in Table S3.

### 2.5 SARS‐CoV‐2 Spike‐Mediated Pseudovirus Entry Assay

HepG2 and Huh7 cells were seeded in 96‐well plates (10,000 cells per well) and cultured with 5, 25 and 100 mM glucose for 12 h, respectively, prior to the transduction of VSV‐SARS‐2‐S‐luc pseudovirus (Delivectory Biosciences, no. SPIKEY‐EL). In cellulo pseudovirus transduction was conducted as described previously [4], and transduction efficiency was analysed by quantifying the activity of *Renilla* luciferase in cell lysates using the ONE‐Glo Luciferase Assay (Promega, no. E1960). For experiments involving glucose‐lowering drugs, hepatic cells were cultured with dapagliflozin (0.5 mM), exenatide (100 nM), metformin (50 mM) or sitagliptin (600 nM) under 100 mM glucose conditions for 6 h before pseudovirus transduction, and then transduction efficiency was determined after 48 h incubation.

### 2.6 Statistical Analysis

All data represented at least three independent experiments and were shown as mean ± SEM. Statistical analysis was performed with GraphPad Prism 8. Two‐tailed Student's *t*‐test was used to determine the difference between two independent groups, with *p* < 0.05 considered significant.

For further details regarding the materials and methods used, refer to the Supporting Information.

## 3 Results

### 3.1 High Glucose Induces Expression of SARS‐CoV‐2 Entry Factors

Hyperglycaemia, hyperinsulinaemia and hyperlipidaemia, which are coordinately regulated by various organs and tissues, such as the liver and pancreas, represent the most common metabolic risk factors for COVID‐19 comorbidities [23, 25, 26, 27]. Although SARS‐CoV‐2 primarily targets the upper respiratory tract, in severe cases the lungs, recent evidence demonstrates that it may directly infect multiple other organs, including the liver and pancreas [28, 29, 30, 31, 32, 33, 34]. We thus assessed the potential effects of COVID‐19‐related metabolic stresses on the expression of SARS‐CoV‐2 entry factors in the lung, liver and pancreatic cells.

We treated human lung (A549 and H1299), hepatic (HepG2 and Huh7) and pancreatic (MIA PaCa‐2 and Panc‐1) cells with 10 nM insulin (equivalent to 1390 mU/L), a concentration that is much higher than the postprandial level (100–200 mU/L) and is widely used for mimicking insulin stimulation in vitro [13, 18, 23, 35], and then measured changes in levels of critical entry factors for SARS‐CoV‐2 infection, including *Ace2*, *Tmprss2*, *Tmprss4*, *Furin* and *Nrp1*. qPCR analysis revealed that mRNA levels of these entry factors were almost unchanged in response to insulin stimulation (Figure S1). Western blotting confirmed levels of these proteins remained unchanged upon insulin stimulation (Figure S1).

To mimic a high lipid environment, cells were treated with 250 μM palmitic acid (PA) for 24 h before determining gene expression without affecting cell viability [36]. Similarly, neither mRNA nor protein levels of these host factors were obviously and consistently affected by PA treatment (Figure S2), indicating a minor effect of hyperlipidaemia on SARS‐CoV‐2 host factors.

**FIGURE 1:**
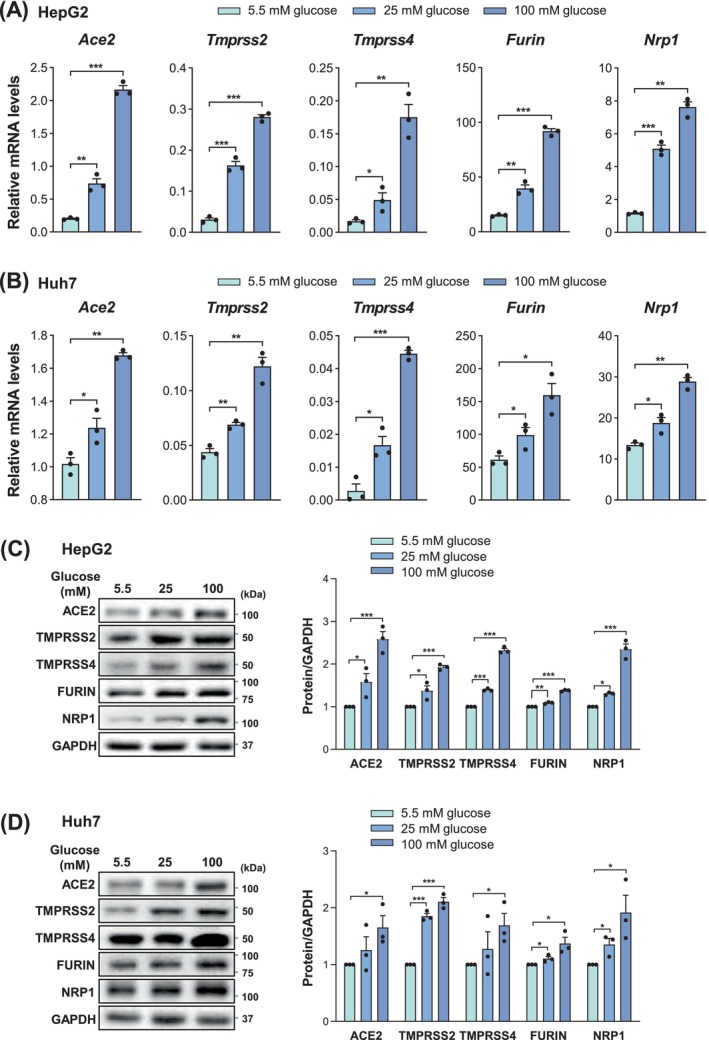
High glucose induces the expression of SARS‐CoV‐2 entry factors. (A, B) mRNA levels of SARS‐CoV‐2 entry factors under 5.5, 25 and 100 mM glucose, respectively, for 48 h in HepG2 (A) and Huh7 (B) cells. (C, D) Protein levels of SARS‐CoV‐2 entry factors under 5.5, 25 and 100 mM glucose, respectively, for 48 h in HepG2 (C) and Huh7 (D) cells. Data are shown as mean ± SEM. *p < 0.05, **p < 0.01, ***p < 0.001 (two‐tailed Student's *t*‐test).

Next, cells were cultured in normal (5.5 mM, equivalent to 0.99 g/L) and high glucose conditions (25 and 100 mM, equivalent to 4.5 and 18 g/L, respectively), which have been widely used to mimic human physiological and pathological (mild and severe hyperglycaemia) conditions, respectively [21, 37]. Protein levels of SARS‐CoV‐2 host factors remained unchanged with elevated glucose levels in the lung cells (A549 and H1299) (Figure S3A–D). In the pancreatic cells, their expression exhibited an obvious tendency toward an increase in high glucose conditions, although there were certain discrepancies between MIA Paca‐2 and Panc‐1 cell lines (Figure S3E–H). Intriguingly, their mRNAs were significantly and consistently increased in the hepatic (HepG2 and Huh7) cells under high glucose conditions (Figure 1A,B), and elevation of their proteins was verified by western blotting (Figure 1C,D).

Taken together, these results suggest that hyperglycaemia, but not hyperinsulinaemia or hyperlipidaemia, might participate in the regulation of SARS‐CoV‐2 host factors and virus infection, particularly in liver cells.

### 3.2 mTOR Might Partially Mediate High Glucose‐Induced Expression of SARS‐CoV‐2 Entry Factors

**FIGURE 2:**
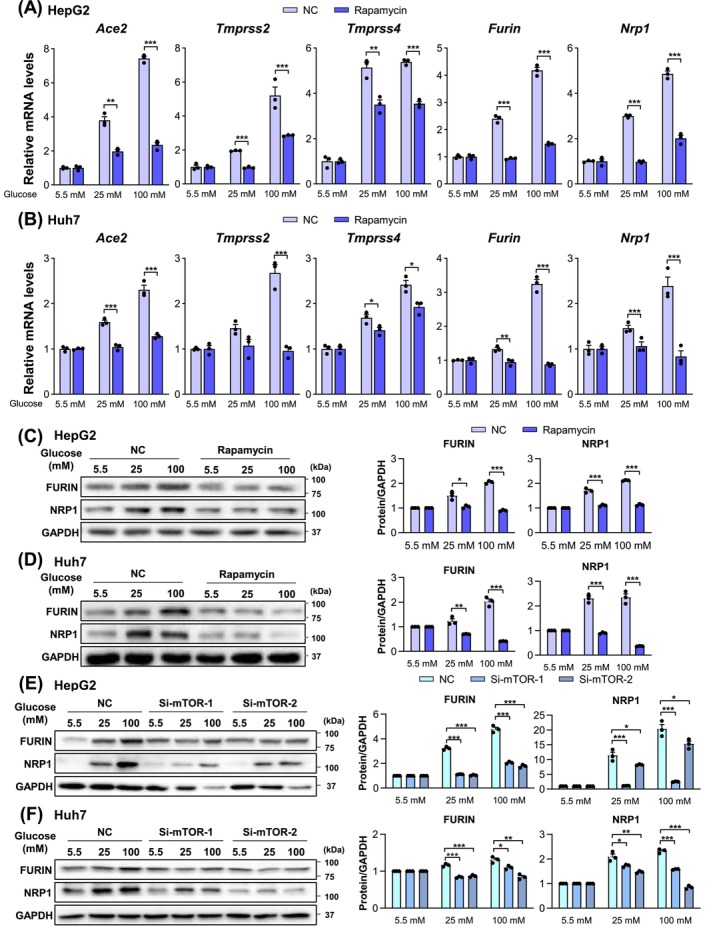
mTOR mediates high glucose‐induced expressions of SARS‐CoV‐2 entry factors. (A, B) mRNA levels of SARS‐CoV‐2 entry factors in HepG2 (A) and Huh7 (B) cells treated with rapamycin (2 μM) for 24 h under indicated glucose concentrations. (C, D) Protein levels of SARS‐CoV‐2 entry factors (NRP1 and FURIN) in HepG2 (C) and Huh7 (D) cells treated with rapamycin (2 μM) for 24 h under indicated glucose concentrations. (E, F) Protein levels of SARS‐CoV‐2 entry factors (NRP1 and FURIN) in HepG2 (E) and Huh7 (F) cells treated with mTOR siRNAs for 48 h under indicated glucose concentrations. Data are shown as mean ± SEM. *p < 0.05, **p < 0.01, ***p < 0.001 (two‐tailed Student's *t*‐test).

The mTOR pathway serves as a nexus for linking nutrient excess including glucose, metabolism and disease [38]. We thus tested if mTOR might mediate high glucose‐induced SARS‐CoV‐2 host factor expression. To this end, HepG2 and Huh7 cells were incubated with rapamycin, a mTOR inhibitor, under different glucose concentrations. Notably, rapamycin treatment led to a decrease in the mRNA levels of SARS‐CoV‐2 host factors under high glucose conditions (25 and 100 mM) (Figure 2A,B). Moreover, this suppressive effect on protein levels was confirmed by western blotting (Figure 2C,D). Consistently, knockdown of mTOR by small interfering RNAs (siRNAs) inhibited high glucose‐induced expression of FURIN and NRP1 in both HepG2 and Huh7 cells (Figure 2E,F).

Together, these results suggest that mTOR may mediate high glucose‐induced SARS‐CoV‐2 host factor expression.

### 3.3 High Glucose Promotes SARS‐CoV‐2 Infection

**FIGURE 3:**
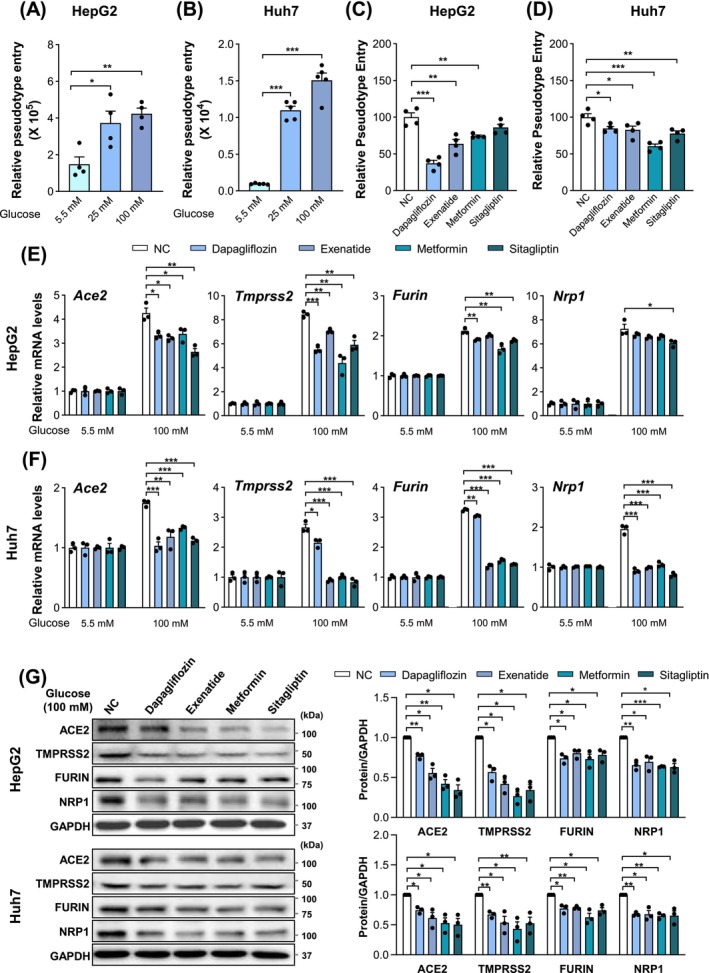
High glucose promotes SARS‐CoV‐2 pseudoviruses entry in vitro. (A, B) SARS‐CoV‐2 pseudoviruses entry efficiency of HepG2 (A) and Huh7 (B) cells under indicated glucose concentrations. Cells were preincubated with the indicated glucose concentrations for 12 h, and subsequently inoculated with VSV‐SARS‐2‐S‐luc. Forty‐eight hours later, pseudovirus entry was measured by analysing luciferase activity in cell lysates. (C, D) SARS‐CoV‐2 pseudoviruses entry efficiency of HepG2 (C) and Huh7 (D) cells with dapagliflozin, exenatide, metformin or sitagliptin treatment. Cells were preincubated with the indicated glucose‐lowering drugs for 6 h prior to pseudovirus inoculation. (E, F) mRNA levels of SARS‐CoV‐2 entry factors treated with glucose‐lowering drugs for 24 h under indicated glucose conditions in HepG2 (E) and Huh7 (F) cells. (G) Protein levels of SARS‐CoV‐2 entry factors treated with glucose‐lowering drugs for 24 h under 100 mM glucose conditions in HepG2 and Huh7 cells. Data are shown as mean ± SEM. *p < 0.05, **p < 0.01, ***p < 0.001 (two‐tailed Student's *t*‐test).

Given the close association between the levels of host factors and SARS‐CoV‐2 infection [4], we hypothesized that pathological glucose concentrations may enable cells susceptible to SARS‐CoV‐2 infection. To test this idea, we used a recently developed pseudovirus system, in which replication‐defective vesicular stomatitis virus (VSV) particles were pseudotyped with SARS‐CoV‐2 spike protein (SARS‐CoV‐2‐S‐luc), and monitored the viral entry step by quantifying the luciferase *Renilla* activity [4]. It has previously been shown that a broad spectrum of human cell lines can be infected by VSV particles [39]. In line with this, SARS‐CoV‐2‐S‐luc was able to enter both HepG2 and Huh7 cells under physiological glucose condition (5.5 mM), although the susceptibility to infection was much less in Huh7 cells (Figure 3A,B). Intriguingly, pathological glucose concentrations (25 and 100 mM) resulted in about a 2.5‐fold increase in pseudovirus entry efficiency in HepG2 cells, and this increase was augmented in Huh7 cells, leading to over 10‐fold greater than that of normal glucose levels (5.5 mM) (Figure 3A,B). These results strongly suggest that pathological glucose levels may enhance the hepatic infection of SARS‐CoV‐2.

### 3.4 Glucose‐Lowering Drugs Reduce SARS‐CoV‐2 Infection

Hyperglycaemia and type 2 diabetes are highly prevalent medical conditions in hospitalised COVID‐19 patients and are strongly associated with worse outcomes, while glucose‐lowering drugs are commonly used to combat these diseases [40]. Therefore, we evaluated the possible effect of glucose‐lowering agents on SARS‐CoV‐2 infection.

Four representative glucose‐lowering drugs by targeting distinct molecules and pathways, namely metformin (the first‐line drug), dapagliflozin (a SGLT2 inhibitor), sitagliptin (a DDP4 inhibitor) and exenatide (a GLP‐1 receptor agonist), were chosen for investigation. HepG2 and Huh7 cells were cultured with pathological glucose concentrations (100 mM) and then treated with different glucose‐lowering drugs, respectively. The luciferase activity showed that dapagliflozin (0.5 mM), exenatide (100 mM) and metformin (50 mM) significantly reduced pseudovirus entry efficiency in both HepG2 and Huh7 cells (Figure 3C,D). Sitagliptin (600 nM) resulted in a significant decrease in Huh7 cells, but not in HepG2 cells. Additionally, dapagliflozin and metformin appeared to have the most inhibition on pseudovirus entry in HepG2 and Huh7 cells, respectively. These results collectively suggest that glucose‐lowering drugs may prevent SARS‐CoV‐2 infection under hyperglycaemia conditions, albeit with differing efficacy in a cell‐dependent manner.

Given the positive correlation between hyperglycaemia and SARS‐CoV‐2 host factor expression, as well as the negative association between glucose‐lowering drugs and SARS‐CoV‐2 entry, it is reasonable to postulate that these glucose‐lowering therapies might repress the expression of SARS‐CoV‐2 host factors. In line with this notion, these four glucose‐lowering drugs generally suppressed the mRNA levels of SARS‐CoV‐2 host factors (*Ace2*, *Tmprss2*, *Furin* and *Nrp1*) in both HepG2 and Huh7 cells under pathological glucose conditions (100 mM), but not under physiological glucose conditions (5.5 mM) (Figure 3E,F). Western blotting further confirmed this inhibitory effect on the protein level of host factors (Figure 3G). Of note, the inhibitory effect of these glucose‐lowering drugs on both mRNA and protein levels of SARS‐CoV‐2 host factors varied in HepG2 and Huh7 cells (Figure 3E–G), further supporting a cell‐dependent regulation.

Taken together, these data suggest that glucose‐lowering drugs could reduce the enhancement of SARS‐CoV‐2 infection induced by hyperglycaemia through decreasing the expression of host factors.

### 3.5 SARS‐CoV‐2 Entry Factors Are Induced in the Livers of NAFLD and Diabetes

**FIGURE 4:**
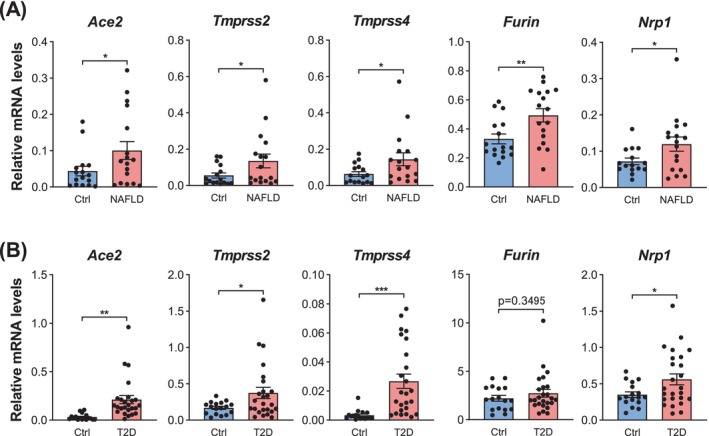
SARS‐CoV‐2 entry factors are induced in the livers of NAFLD and diabetes patients. (A) mRNA levels of SARS‐CoV‐2 entry factor expression in the livers of NAFLD patients (*n* = 17) and healthy controls (*n* = 16). (B) mRNA levels of SARS‐CoV‐2 entry factor expression in the livers of patients with type 2 diabetes (*n* = 25) and healthy controls (*n* = 17). Data are shown as mean ± SEM. *p < 0.05, **p < 0.01, ***p < 0.001 (two‐tailed Student's *t*‐test). T2D, type 2 diabetes.

Hyperglycaemia is closely associated with obesity, NAFLD and type 2 diabetes [12, 41, 42]. Thus, we examined the expression of SARS‐CoV‐2 host factors in the livers of patients with NAFLD. qPCR analyses revealed that SARS‐CoV‐2 entry factors, including *Ace2*, *Tmprss2*, *Tmprss4*, *Furin* and *Nrp1*, were significantly induced in the livers of NAFLD patients (Figure 4A). Moreover, these factors, except *Furin*, were also upregulated in the livers of patients with type 2 diabetes (Figure 4B), further supporting a positive link between hyperglycaemia‐related diseases and the expression of SARS‐CoV‐2 host factors.

## 4 Discussion

In this study, we report a role of hyperglycaemia in SARS‐CoV‐2 infection. We found that hyperglycaemia, but not hyperinsulinaemia or hyperlipidaemia, led to an increase in the expression of SARS‐CoV‐2 entry factors and promoted SARS‐CoV‐2 entry into hepatic cells. These results established for the first time, to our knowledge, a link between a metabolic risk factor, host factor expression and SARS‐CoV‐2 infection. Furthermore, we showed that classic glucose‐lowering drugs were able to reduce SARS‐CoV‐2 infection in cultured hepatocytes under pathological glucose levels. These findings not only uncover a metabolic mechanism underlying SARS‐CoV‐2 entry but also provide useful information regarding the clinical application of glucose‐lowering therapies in COVID‐19 patients under comorbid hyperglycaemia conditions.

Hyperglycaemia, hyperinsulinaemia and hyperlipidaemia, representative characteristics of many metabolic disorders including type 2 diabetes, obesity and cardiovascular diseases, are the most common risk factors for severe COVID‐19 complications [23, 25]. These correlations have engendered great interest, and tremendous progress has been made in understanding the metabolic aspects of SARS‐CoV‐2‐triggered pathophysiology, as well as the contribution of preexisting aberrant metabolism to COVID‐19 severity [8, 25, 43, 44]. However, the effect of COVID‐associated metabolic risk factors on SARS‐CoV‐2 infection is poorly understood. In the present study, we first screened the expression of SARS‐CoV‐2 entry factors in lung, hepatic and pancreatic cells under distinct metabolic stress conditions. These analyses revealed that pathological glucose levels, rather than excess insulin or lipids, specifically induced the expression of entry factors (*Ace2*, *Tmprss2*, *Tmprss4* and *Nrp1*) in hepatic cells, but not in lung cells. Interestingly, high glucose levels could also result in an increase in the expressions of entry factors in pancreatic cells, albeit the statistical significance and the consistency in different cell lines were less than that of hepatic cells. These observations are concordant with the fact that both the liver and pancreas, but not the lung, are critical metabolic organs in response to glucose and play crucial roles in systemic metabolic homeostasis [26, 41]. Importantly, pathological glucose levels can markedly promote SARS‐CoV‐2 entry to cultured liver cells, in agreement with their induction on entry factor expression, as well as the positive correlation between entry factor levels and SARS‐CoV‐2 infection as previously reported [2, 4]. Of note, although growing evidence supports that SARS‐CoV‐2 could directly infect the liver tissues, it remains a topic of debate [28, 34, 45, 46, 47, 48, 49, 50]. Here we found that the hepatic SARS‐CoV‐2 infectivity was relatively low under physiological glucose levels, but noticeably increased under pathological glucose levels. Moreover, different hepatic cell lines, such as HepG2 and Huh7, appeared to have varied susceptibility to SARS‐CoV‐2 infection. These observations not only support the hepatic infection of SARS‐CoV‐2, but also suggest a tangible explanation for the heterogeneity of hepatic SARS‐CoV‐2 susceptibility as described previously [28, 34, 45, 46, 47, 48, 49, 50], which might associate with different metabolic statuses. In addition, it is important to note that some protein levels, such as FURIN, in response to glucose treatment, showed significant, but slight changes (Figure 1C,D), although their mRNA changes were more obvious (Figure 1A,B), possibly due to posttranscriptional regulation. It is of interest for future study to verify the responsiveness of SARS‐CoV‐2 host factors to high glucose levels and hyperglycaemia‐induced SARS‐CoV‐2 infection in additional in vitro and in vivo models, such as organoids, animals and clinical specimens.

Hyperglycaemia is an independent risk factor for COVID‐19 severity and mortality [51, 52]. Meanwhile, COVID‐19 might predispose infected individuals to new‐onset hyperglycaemia [53, 54]. This devastating feedback circuitry, involving hyperglycaemia and COVID‐19, emphasises the use of glucose‐lowering medications for combating the COVID‐19 pandemic. Indeed, numerous studies suggest that glucose‐lowering agents may have beneficial (i.e., metformin and meglitinides) or undesirable (such as insulin) effects on COVID‐related mortality, albeit the conclusions are confounding and should be interpreted with caution, in view of the syndromic nature of COVID comorbidities, such as type 2 diabetes, as well as the use of different medications at distinct disease stages [40, 55, 56]. On the other hand, the link of obesity and diabetes, classical metabolic disorders with hyperglycaemia, to SARS‐CoV‐2 infection remains unclear till now, due to the noticeable variations in the prevalence of obesity and diabetes in COVID‐19 patients [25, 43]. In this study, we revealed an inhibitory effect of glucose‐lowering drugs that we examined on SARS‐CoV‐2 entry into hepatic cells under pathological glucose conditions. However, this inhibition is likely unable to counteract the hyperglycaemia‐elicited increase of SARS‐CoV‐2 infection. These results suggest that individuals with hyperglycaemia might be susceptible to SARS‐CoV‐2 infection, which awaits further investigation. In addition, our results demonstrated that both hyperglycaemia and glucose‐lowering drugs affect hepatic SARS‐CoV‐2 infection through modulating host factors, but not the virus per se. Thus, it is conceivable that they might also have a similar role in the infection of SARS‐CoV‐2 variants, including Omicron, which warrants future investigation in cell, animal and preclinical studies.

In summary, this study uncovers a previously unknown link between hyperglycaemia and SARS‐CoV‐2 infection, suggesting a metabolic mechanism underlying COVID‐19 pathogenesis and promising strategies for preventing this disastrous pandemic.

## Author Contributions

**Guocheng Rao:** investigation (lead), methodology (lead), writing – original draft (lead). **Xiongbo Sang:** conceptualization (equal), methodology (equal), writing – original draft (equal). **Xinyue Zhu:** investigation (equal), supervision (equal), writing – original draft (equal). **Sailan Zou:** formal analysis (equal), software (equal). **Yanyan Zhang:** formal analysis (equal), software (equal). **Wei Cheng:** data curation (equal). **Yan Tian:** methodology (equal), visualization (equal). **Xianghui Fu:** conceptualization (lead), project administration (lead), resources (lead), writing – review and editing (lead).

## Disclosure

Permission to reproduce material from other sources: No materials reproduced from other sources are included in this manuscript.

## Ethics Statement

The study was performed in accordance with the principles of the Declaration of Helsinki and approved by the Institutional Review Board and Biomedical Ethics Committee of West China Hospital of Sichuan University (WCH/SCU).

## Consent

Fully informed consent was obtained from all patients.

## Conflicts of Interest

The authors declare no conflicts of interest.

## Supporting information


Data S1.

## Data Availability

All data presented are available upon request.
