# Supplementary material for: Pathological Glucose Levels Enhance Entry Factor Expression and Hepatic SARS‐CoV‐2 Infection
Source: J Cell Mol Med. 2025 May 29;29(11):e70581. doi: 10.1111/jcmm.70581 (PMC12122388; doi:10.1111/jcmm.70581)
Supplement: Supplementary file 1 — Data S1. [file JCMM-29-e70581-s001.zip › jcmm70581-sup-0001-Supinfo.docx]

**Pathological Glucose Levels Enhance Entry Factor Expression and Hepatic SARS-CoV-2 Infection**

Guocheng Rao, Xiongbo Sang, Xinyue Zhu, Sailan Zou, Yanyan Zhang, Wei Cheng, Yan Tian, Xianghui Fu

**﻿Supplementary Material of contents**

Figure S1﻿ – Expressions of SARS-CoV-2 entry factors in insulin-treated cells

Figure S2﻿ – Expressions of SARS-CoV-2 entry factors in palmitic acid-treated cells

Figure S3﻿ – Expressions of SARS-CoV-2 entry factors in glucose-induced cells

Table S1 – Sequences of siRNAs for mTOR

Table S2 – Sequences of QRT-PCR primers

Table S3 – Antibodies for western blotting

**Figure legends**

**Figure 1. High glucose induces the expression of SARS-CoV-2 entry factors.**

**(A, B)** mRNA levels of SARS-CoV-2 entry factors under 5.5 mM, 25 mM, and 100 mM glucose, respectively, for 48 h in HepG2 **(A)** and Huh7 **(B)** cells.

**(C, D)** Protein levels of SARS-CoV-2 entry factors under 5.5 mM, 25 mM, and 100 mM glucose, respectively, for 48 h in HepG2 **(C)** and Huh7 **(D)** cells.

Data are shown as mean ± SEM. *p < 0.05, ***p < 0.01, ***p < 0.001 (Two-tailed Student’s t test).

**Figure 2. mTOR mediates high glucose-induced expressions of SARS-CoV-2 entry factors.**

**(A, B)** mRNA levels of SARS-CoV-2 entry factors in HepG2 **(A)** and Huh7 **(B)** cells treated with rapamycin (2 μM) for 24 hours under indicated glucose concentrations.

**(C, D)** Protein levels of SARS-CoV-2 entry factors (NRP1 and FURIN) in HepG2 **(C)** and Huh7 **(D)** cells treated with rapamycin (2 μM) for 24 hours under indicated glucose concentrations.

**(E, F)** Protein levels of SARS-CoV-2 entry factors (NRP1 and FURIN) in HepG2 **(E)** and Huh7 **(F)** cells treated with mTOR siRNAs for 48 hours under indicated glucose concentrations.

Data are shown as mean ± SEM. *p < 0.05, ***p < 0.01, ***p < 0.001 (Two-tailed Student’s t test).

**Figure 3. High glucose promotes SARS-CoV-2 pseudoviruses entry *in vitro*.**

**(A, B)** SARS-CoV-2 pseudoviruses entry efficiency of HepG2 **(A)** and Huh7 **(B)** cells under indicated glucose concentrations. Cells were preincubated with the indicated glucose concentrations for 12 hours, and subsequently inoculated with VSV-SARS-2-S-luc. 48 hours later, pseudovirus entry was measured by analyzing luciferase activity in cell lysates.

**(C, D)** SARS-CoV-2 pseudoviruses entry efficiency of HepG2 **(C)** and Huh7 **(D)** cells with dapagliflozin, exenatide, metformin, or sitagliptin treatment. Cells were preincubated with the indicated glucose-lowering drugs for 6 hours prior to pseudovirus inoculation.

**(E, F)** mRNA levels of SARS-CoV-2 entry factors treated with glucose-lowering drugs for 24 hours under indicated glucose conditions in HepG2 **(E)** and Huh7 **(F)** cells.

**(G)** Protein levels of SARS-CoV-2 entry factors treated with glucose-lowering drugs for 24 hours under 100 mM glucose conditions in HepG2 and Huh7 cells.

Data are shown as mean ± SEM. *p < 0.05, ***p < 0.01, ***p < 0.001 (Two-tailed Student’s t test).

**Figure 4. SARS-CoV-2 entry factors are induced in the livers of NAFLD and diabetes patients.**

**(A)** mRNA levels of SARS-CoV-2 entry factor expression in the livers of NAFLD patients (n=17) and healthy controls (n=16).

**(B)** mRNA levels of SARS-CoV-2 entry factor expression in the livers of patients with type 2 diabetes (n=25) and healthy controls (n=17). T2D, type 2 diabetes.

Data are shown as mean ± SEM. *p < 0.05, ***p < 0.01, ***p < 0.001 (Two-tailed Student’s t test).

**Figure S1. Expressions of SARS-CoV-2 entry factors in insulin-treated cells.**

**(A-D)** mRNA and protein levels of SARS-CoV-2 entry factors in HepG2 **(A, B)** and Huh7 **(C, D)** cells treated with insulin (10 nM) for 12 hours.

**(E-H)** mRNA and protein levels of SARS-CoV-2 entry factors in H1299 **(E, F)** and A549 **(G, H)** cells treated with insulin (10 nM) for 12 hours.

**(I-L)** mRNA and protein levels of SARS-CoV-2 entry factors in MIA PaCa-2 **(I, J)** and PANC-1 **(K, L)** cells treated with insulin (10 nM) for 12 hours.

Data are shown as mean ± SEM. *p < 0.05, ***p < 0.01, ***p < 0.001 (Two-tailed Student’s t test).

**Figure S2. Expressions of SARS-CoV-2 entry factors in palmitic acid-treated cells.**

**(A-D)** mRNA and protein levels of SARS-CoV-2 entry factors in HepG2 **(A, B)** and Huh7 **(C, D)** cells treated with palmitic acid (250 μM) for 12 hours.

**(E-H)** mRNA and protein levels of SARS-CoV-2 entry factors in H1299 **(E, F)** and A549 **(G, H)** cells treated with palmitic acid (250 μM) for 12 hours.

**(I-L)** mRNA and protein levels of SARS-CoV-2 entry factors in MIA PaCa-2 **(I, J)** and PANC-1 **(K, L)** cells treated with palmitic acid (250 μM) for 12 hours.

Data are shown as mean ± SEM. *p < 0.05, ***p < 0.01, ***p < 0.001 (Two-tailed Student’s t test).

**Figure S3. Expressions of SARS-CoV-2 entry factors in glucose-induced cells.**

**(A, B)** mRNA **(A)** and protein **(B)** levels of SARS-CoV-2 entry factors in H1299 cells under indicated glucose conditions for 48 hours.

**(C, D)** mRNA **(C)** and protein **(D)** levels of SARS-CoV-2 entry factors in A549 cells under indicated glucose conditions for 48 hours.

**(E, F)** mRNA **(E)** and protein **(F)** levels of SARS-CoV-2 entry factors in MIA PaCa-2 cells under indicated glucose conditions for 48 hours.

**(G, H)** mRNA **(G)** and protein **(H)** levels of SARS-CoV-2 entry factors in PANC-1 cells under indicated glucose conditions for 48 hours.

Data are shown as mean ± SEM. *p < 0.05, ***p < 0.01, ***p < 0.001 (Two-tailed Student’s t test).
