# Supplementary material for: Pathological Glucose Levels Enhance Entry Factor Expression and Hepatic SARS‐CoV‐2 Infection
Source: J Cell Mol Med. 2025 May 29;29(11):e70581. doi: 10.1111/jcmm.70581 (PMC12122388; doi:10.1111/jcmm.70581)
Supplement: Supplementary file 1 — Data S1. [file JCMM-29-e70581-s001.zip › jcmm70581-sup-0003-Supplementarytables.docx]

**Table S1 – Sequences of siRNAs for mTOR**

| **Gene** | **Sequence (5’ – 3’)** |
| --- | --- |
| simTOR-1 | GCAGAUUUGCCAACUAUCUTT |
| simTOR-2 | GAAUUCUGGGUCAUGAACATT |

**Table S2 – Sequences of QRT-PCR primers**

| **Gene** | **Forward (5’ – 3’)** | **Reverse (5’ – 3’)** |
| --- | --- | --- |
| ACE2 | CGAAGCCGAAGACCTGTTCTA | GGGCAAGTGTGGACTGTTCC |
| ACTIN | TAGGCACCAGGGTGTGATGG | AGGGCATACCCCTCGTAGAT |
| FURIN | CCTGGTTGCTATGGGTGGTAG | AAGTGGTAATAGTCCCCGAAGA |
| GAPDH | ACAGCCTCAAGATCATCAGCAA | ACCACTGACACGTTGGCAGT |
| NRP1 | TACGAAACACATGGTGCAGGA | AGGGAATCCGGGGGACTTTA |
| TMPRSS2 | GCAGTGGTTTCTTTACGCTGT | CCGCAAATGCCGTCCAATG |
| TMPRSS4 | GACAAACAGCACGTCTGTGGA | GCCTGAGAAAGTGAGTGGGAA |

**Table S3** **– Antibodies for western blotting﻿.**

| **Gene** | **Supplier** | **Cat no.** |
| --- | --- | --- |
| ACE2 | Abcam | ab108252 |
| FURIN | Abcam | ab183495 |
| GAPDH | Proteintech | 60004-1-IG |
| NRP1 | Proteintech | 60067-1- IG |
| TMPRSS2 | Abcam | ab280567 |
| TMPRSS4 | ABclonal | A4865 |
