# Supplementary figures and images for: Pathological Glucose Levels Enhance Entry Factor Expression and Hepatic SARS‐CoV‐2 Infection
Source: J Cell Mol Med. 2025 May 29;29(11):e70581. doi: 10.1111/jcmm.70581 (PMC12122388; doi:10.1111/jcmm.70581)

**Figure S1**

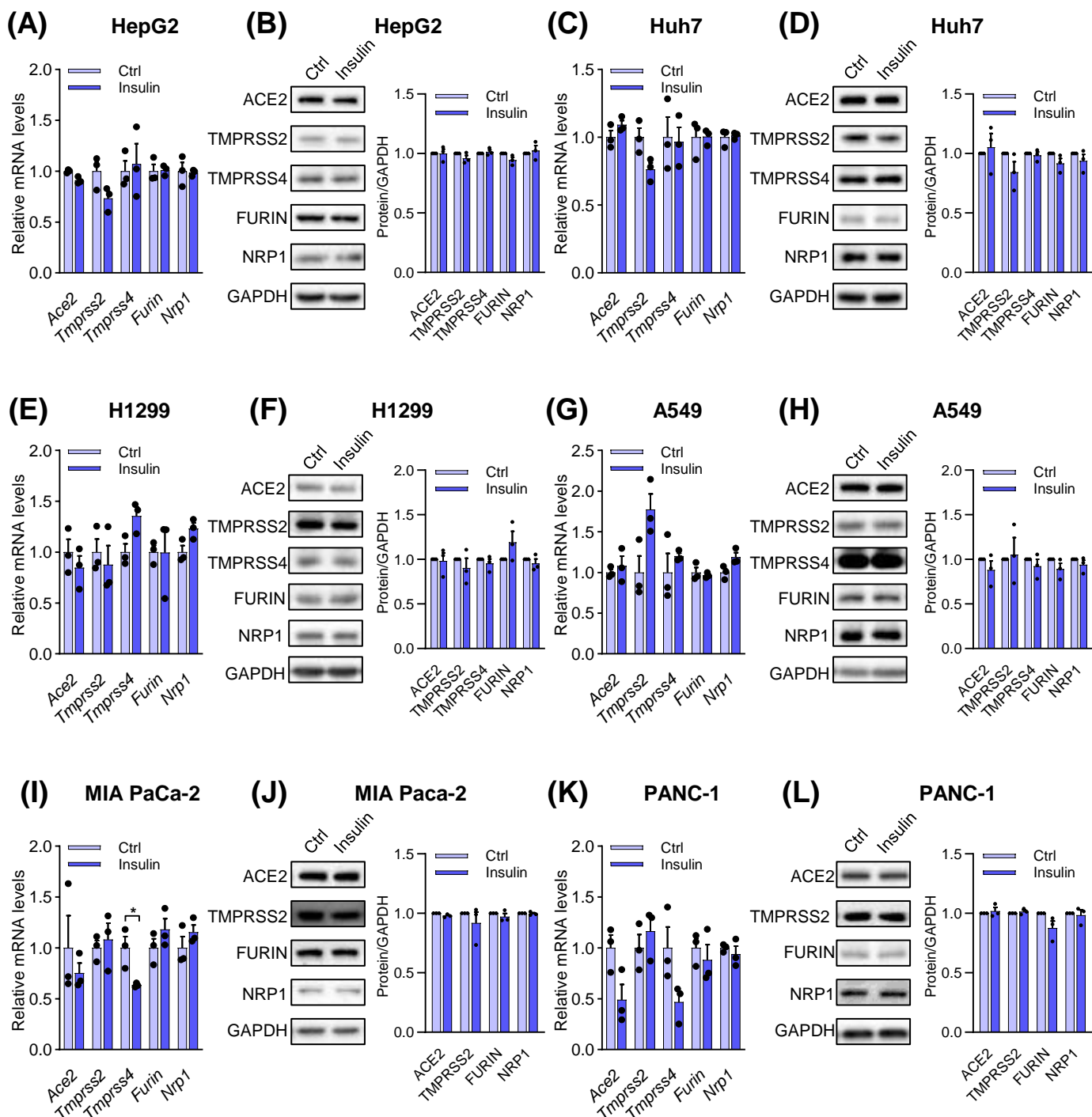

**Figure S2**

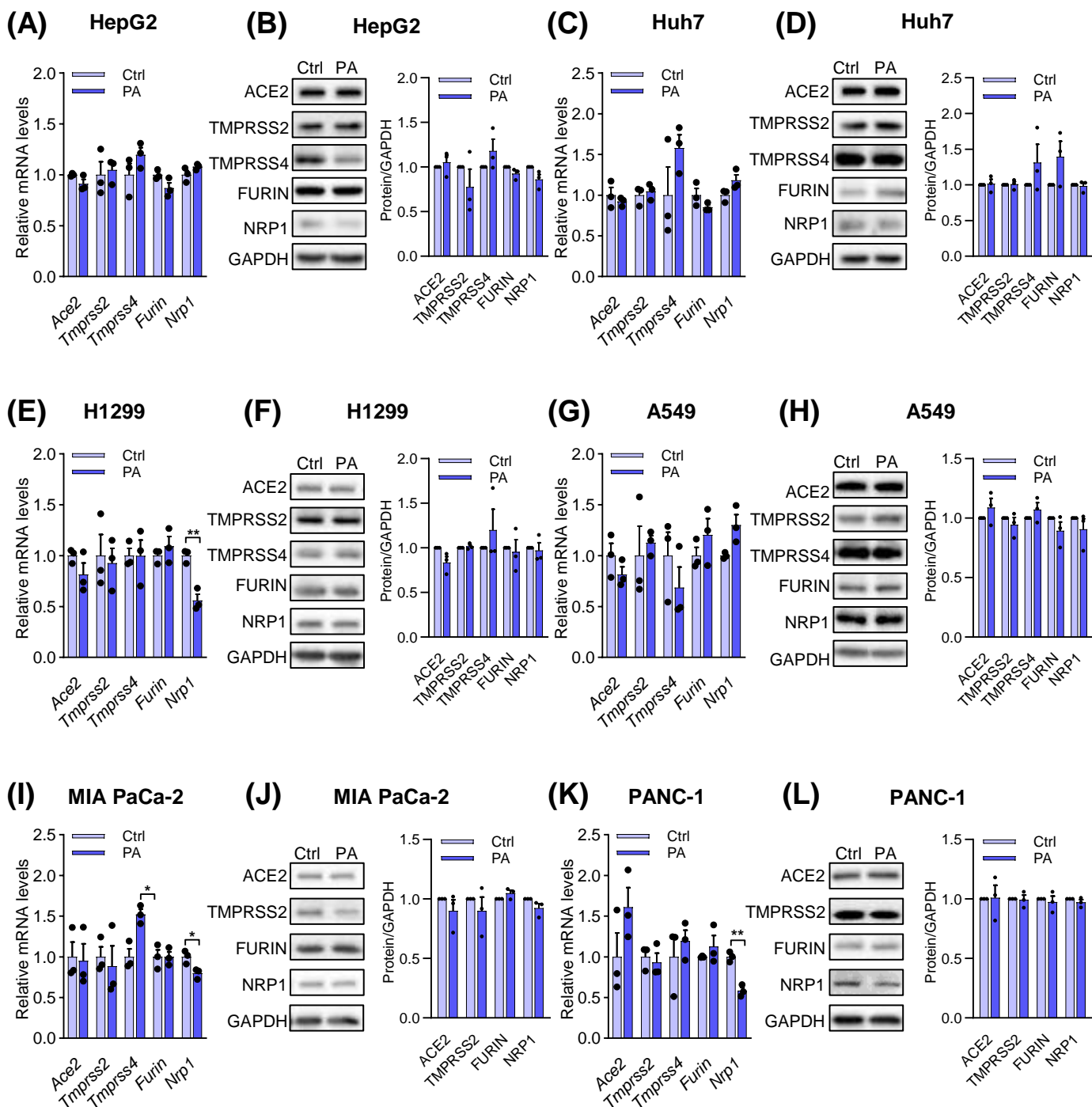

**Figure S3**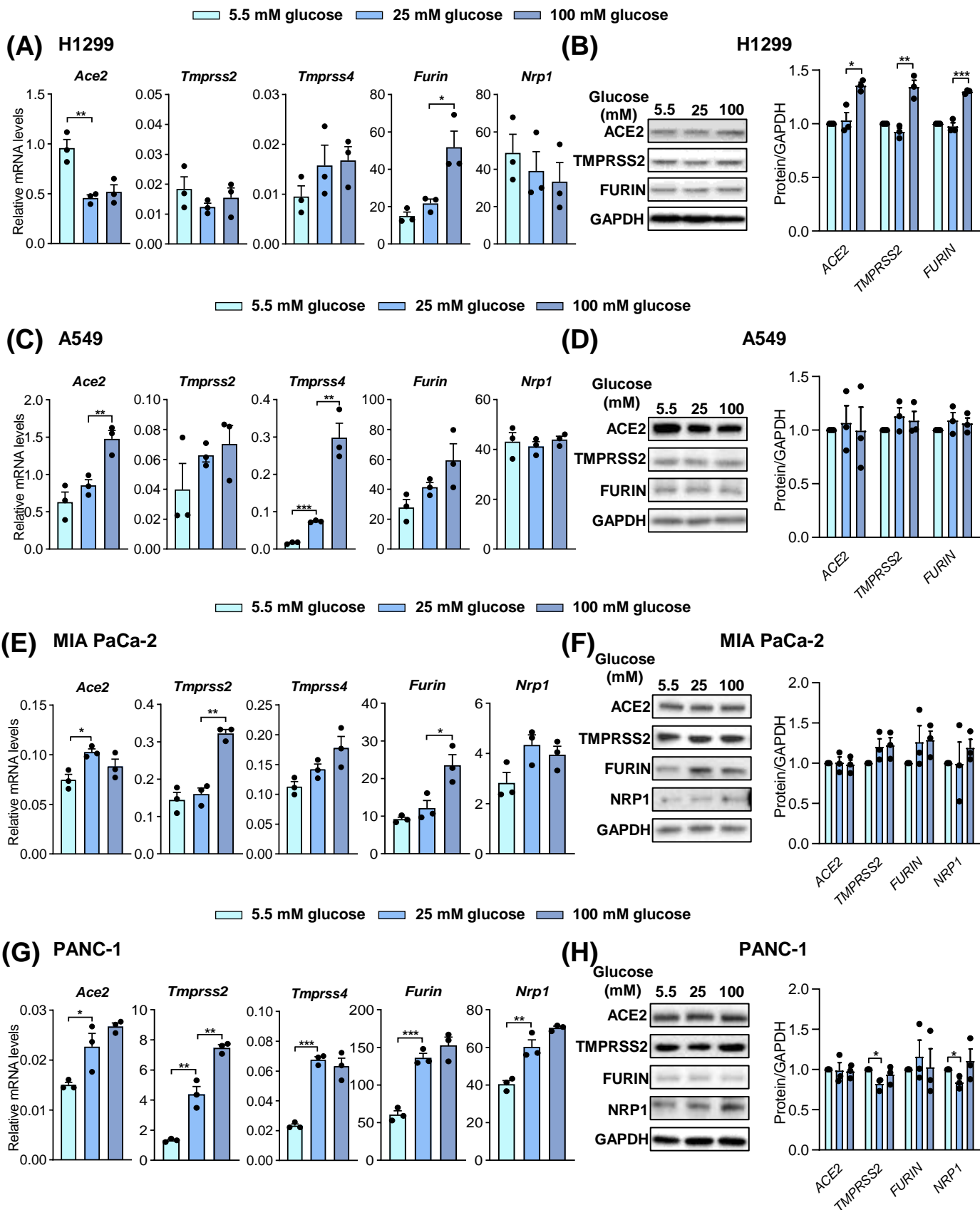

Supplement: Supplementary file 1 — Data S1. [file JCMM-29-e70581-s001.zip › jcmm70581-sup-0002-Supplementaryfigures.pdf]
